# Effectiveness of Acceptance and Commitment Therapy on Interpersonal Problemsand Psychological Flexibility in Female High School Students With Social Anxiety Disorder

**DOI:** 10.5539/gjhs.v8n3p131

**Published:** 2015-07-13

**Authors:** Sayedeh Monireh Azadeh, Hamid Kazemi-Zahrani, Mohammad Ali Besharat

**Affiliations:** 1 Department of Psychology, Faculty of Humanities, Najafabad Branch, Islamic Azad University, Najafabad, Isfahan, Iran; 2 Department of Psychology, Payame Noor University, Tehran, Iran; 3 Department of Psychology, University of Tehran, Tehran, Iran

**Keywords:** acceptance and commitment therapy, interpersonal problems, psychological flexibility, social anxiety disorder

## Abstract

Social anxiety is a psychological disorder which has devastative and pernicious effects on interpersonal relationships and one's psychological flexibility. The aim of this research was to determine the effectiveness of Acceptance and Commitment Therapy on interpersonal problems and psychological flexibility in female high school students with social anxiety disorder. With a semi-experimental design, the subjects were assessed using the Social Anxiety Scale and clinical interview. The statistical population of the research was high school female students studying in 5 areas of Isfahan. 30 individuals were purposively selected as the sample. The subjects of the research were randomly assigned to the experimental and control groups. Acceptance and Commitment Therapy was given in 10 sessions of 90 minutes in the experimental group and the control group did not receive any treatment. Pre-test and post-test scores of Inventory of Interpersonal Problems, and Acceptance and Action Questionnaire were analyzed using multivariate analysis of variance & the results showed that after the intervention, there was a significant difference between the scores of the subjects in the experimental and control groups. This means that Acceptance and Commitment Therapy can influence interpersonal problems and their six dimensions and psychological flexibility as well.

## 1. Introduction

Social anxiety disorder is characterized by the main symptom of a given and persistent fear or anxiety in certain situations in which a person may be scrutinized by others precisely such as being in a group or community, being observed, or giving a speech in front of others (APA, 2013). It is one of the most common disorders among chronic psychological disorders (Andrews, Henderson, & Hall, 2001; McEvoy, Grove, & Slade, 2011) that is seen in almost 13% of people living in the community (Kessler, Petukhova, Sampson, Zaslavsky, & Wittchen, 2012) and has many devastating effects on one's job, education, interpersonal functioning or performance (Hofmann & Otto, 2008), body and personal life (Ruscio et al., 2008; Furmark, 2002) and uniquely predicted by anxiety sensitivity social concerns (Carter, Sbrocco, & Avati, 2009; Thibodeau, Gomez-Perez, & Asmundson, 2012; Olthuis, Watt, & Stewart, 2014). Social anxiety disorder is one of the early or premature anxiety disorders (Andrews, Henderson, & Hall, 2001; McEvoy, Grove, & Slade, 2011). Its onset is of adolescence and women are more susceptible to it than men (Ledley & Heimberg, 2006).

One of the important aspects of a person's life that is strongly associated with psychological distressesis interpersonal problems (Olivares, Piqueras, & Rosa, 2006). Interpersonal problems are defined as problems that are repeated in one's life and an individual experiences them in relation to others (Leary, 1957; Horowitz, 1994). They can be briefly divided into six areas including assertiveness, sociability, submissiveness, intimacy, taking responsibility, and controlling (Horowitz, Rosenberg, Ureno, & Villasenor, 1988). Interpersonal relationships are so important that a special share or contribution is considered for their role in the constitution and persistence of psychological disorders in valid pathological and diagnostic classifications (APA, 2000). People with social anxiety experience limited and damaged interpersonal relationships (Wittchen, Stein, & Kessler, 1999). Therefore, the symptoms of social anxiety can be considered as part of a general interpersonal trauma (Tavali, Allahyary, & Azad Fallah, 2012).

On the other hand, what is considered as a key factor in social anxiety disorder (Biglan, Hayes, & Pistorello, 2008) and leads to the continuation or persistence and intensification of the disorder is lack of psychological flexibility which is described as dominance in verbal judgments and adjustable feelings and coping strategies (eg. avoidance and suppression) which are the cores of human suffering (Masuda et al., 2011). Psychological flexibility is in fact numerous behavioral patterns obtained by an individual's experiences which allow him to act or behave based on what is important to him in life and increase his behavioral flexibility (Bluett, Homan, Morrison, Levin, & Twohig, 2014). Lack of psychological flexibility can be observed in disorders such as depression (Bond & Bunce, 2006; Bohlmeijer, Fledderus, Rokx, & Pieterse, 2011; Forman et al., 2012) and the continuum or spectrum of anxiety disorders (Kashdan, Barrios, Forsyth, & Steger, 2006; Dalrymple & Herbert, 2007).

Among treatments for anxiety disorders, cognitive behavioral therapy (Goldin et al., 2014; McAleavey, Castonguay, & Goldfried, 2014; Boswell et al., 2013), interpersonal therapy (Borge et al., 2008), and exposure (Dixon, Kemp, Farrell, Blakey, & Deacon, 2015; Price, Mehta, Tone, & Anderson, 2011) can be mentioned. Another treatment that has been applied for this disorder is ACT (Dindo, 2015; Roemer, Orsillo, & Salters-Pedneault, 2008; Twohig, Hayes, & Masuda, 2006; Eifert & Forsyth, 2005). ACT is one of the third wave treatments and is considered as a form of cognitive behavioral therapy (Hayes, 2004) which has its roots in the new theory of cognition and language, which is known as the theory of the framework of mental relations (Twohig, Woidneck, & Crosby, 2013). One of the key features of ACT is its pragmatic philosophical structure which focuses on psychological behavior and is presented as pragmatic contextualization (Hayes, Strosahl, & Wilson, 2012). In this therapy, it is presumed that humans find a lot of their feelings, emotions, and internal thoughts annoying and always try to change these internal experiences or to get rid of them (Hayes, Villatte, Levin, & Hildebrandt, 2011). This effort to control is ineffective and in contrast leads to the intensification of feelings, emotions and thoughts that the person at first tried to avoid them (Hayes, Orsillo, & Roemer, 2010). ACT is a treatment approach including six specific psychological processes: acceptance, defusion, self as context, contact with present moment, values, and committed action (Luoma, Hayes, & Walser, 2007). All of these six processes are used with metaphor, experiential exercises and logical contradiction to escape from the literal or verbal content of the language and to communicate more with the continuous flow of experience at the present moment (Twohig, 2012). In fact, this therapy aims to draw the person with social anxiety disorder who is strictly trapped and stuck in experiential avoidance and cognitive fusion into acceptance and defusion.

Considering the use of this new approach for social anxiety disorder and the early onset of the disorder in adolescence, and on the other hand considering the point that the issue of interpersonal problems and psychological flexibility in social anxiety disorder have not been dealt with thoroughly, for better prognosis, and lower health care costs in case of treatment in this period of life, the main goal of the present research was to determine the effectiveness of ACT on interpersonal problems and psychological flexibility of girls with social anxiety disorder.

## 2. Materials and Method

### 2.1 Research Design

This research is quasi-experimental in which the pretest-posttest control group design was used. The treatment is the independent variable and the levels of the treatment are based on acceptance and commitment. Interpersonal problems and psychological flexibility are considered as dependent variables.

**Research Subjects**

The population of this research consisted of all high school girls in Isfahan city in 2014. The sampling was done in two stages: in the first stage, 170 students were selected by convenience method. Students who got high scores in Social Anxiety Scale for Adolescents were identified and were clinically interviewed (according to the criteria of the Diagnostic Statistical Manual, Fifth Edition). In the second stage, 30 students with social anxiety disorder were randomly assigned to two groups as follows: 15 individuals were put in the experimental group with ACT and 15 were assigned in the control group. The criteria for entry to the experimental group were: studying in high school, not taking psychiatric drugs, not having other psychological and personality disorders (determined with clinical interview), not participating simultaneously in other therapy programs and not receiving individual or personal counseling.

**Instruments**

*Social Anxiety Scale for Adolescents*: It includes 28 questions which measures adolescents’ worries, fears, and avoidance behaviors in social situations such as interactions with friends and interaction at school. This scale includes 2 subscales of perception and fear from negative evaluations (15 questions) and tension and inhibition about social contact (13 questions). Each question is answered based on a 5-degree scale.

Alpha coefficients for the subscales of perception and fear from negative evaluations and tension and inhibitionabout social contact and the total score of social anxiety were obtained to be between 0.84 to 0.68 which show high levels of internal consistency SASA (Khodaee, Shekary, Paklk, Buttercream, & Toulabi, 2010). Correlation coefficients obtained from pretest-posttest for the subscales of this questionnaire and the total score of SASA were 0/77, 0/71, 0/60 respectively which were acceptable.

*Inventory of Interpersonal Problems (IIP-60)*: This scale includes 60 items which is obtained from and is based on the results 0f 127-item version of this scale in a sample of students (Besharat, 2005). In the exploratory factor analysis, the research by Besharat (2010) confirmed six factors for the Inventory of Interpersonal Problems in addition to the general factor of interpersonal problems. These six factors are assertiveness, sociability, submissiveness, intimacy, taking responsibility, and controlling. Convergence and differential validity of Inventory of Interpersonal Problems were confirmed according to the research by Besharat based on correlation coefficients of the means of subjects’ scores in Inventory of Interpersonal Problems with the indexes of psychological well-being, psychological helplessness, self-respect, and emotional intelligence. The internal consistency of the scale was calculated by Cronbach's alpha coefficients and was approved by correlation coefficients from 0/82 to 0/93. Test-retest reliability of the scale was approved based on the results of the retest by the coefficients from 0/65 to 0/81.

*Acceptance and Action Questionnaire – II (AAQ-II)*: The main mechanism in ACT, which includes the aforementioned six processes, is psychological flexibility. Acceptance and Action Questionnaire evaluates whether ACT achieves the goal of psychological flexibility or not. In fact, this questionnaire is a self-assessment tool to measure psychological flexibility (Hayes, 2004). AAQ-II is a 10-item instrument with a good internal stability (α= 0/87) and test-retest reliability(r=0/80). AAQ-II includes positive and negative questions and is correlated with variables that are theoretically related to it. The high AAQ-II scores predicted mental health (Bond, Hayes, Baer, Carpenter, Guenole, Orcutt, & Zettle, 2011).

ACT based on the work by McKey, Lev, & Skeen (2012) was planned for a period of two and a half months (10 sessions of 90 minutes). Patients were trained once a week in a group session receiving ACT in accordance with the following content: In the first session, the preliminary test related to ten ineffective schemes in relationships was administered and after its completion and scoring, schemes and coping behaviors were explained and discussed and mindfulness exercises were given to clients. In the second session, schemes’ stimulants were explored and individuals were drawn into creative hopelessness which is part of the acceptance process. In the third session, the consequences of coping behaviors were explained. In the fourth session, values in interpersonal relations were stated and identification of barriers while acting based on values was considered as the agenda or instruction. In the fifth session, individuals were trained in and exercised defusion skills. The sixth session focused on describing in place of judgment in interpersonal relationships. In the seventh session, anger was discussed as one of the most common coping behaviors. In the eighth session, emotions and the incompetency and inefficiency of controlling techniques were taught. In the ninth session, the issue of effective interpersonal communication was discussed. In the last session, all the stated discussions in the sessions were summarized and ways to expand action in the scope or realm of values were dealt with.

### 2.2 Research Procedure

After identifying female students with social anxiety disorder and getting their volunteer satisfaction for participating in the therapy, among subjects, 15 individuals were assigned to the experimental group and 15 individuals were assigned to the control group. Then, both groups completed Inventory of Interpersonal Problems and Acceptance and Action Questionnaire in the pretest stage. The subjects in the experimental group received ACT in 10 90-minute sessions once a week, but the control group did not receive any intervention. At the end of treatment, the subjects in both groups completed the questionnaires again in the post-test stage and finally the obtained data was analyzed by covariance analysis method.

## 3. Research Results

The mean and standard deviation of age was 15.43 and 0.78 with the range of 15 to 16 years old. There was no significant difference between the experimental and control group regarding age and grade variables. In Table 1, the mean and standard deviation of the scores of variables in post-test and pre-test in the experimental and control groups are presented.

**Table 1:** Mean and standard deviation of the experimental and control groups in the studied variables in pre-test and post-test

Situation	The experimental group				The control group			
	
	Post-test		Pre-test		Post-test		Pre-test	
**Variables**	SD	x̄	SD	x̄	SD	x̄	SD	x̄
**Problem with Assertiveness**	0.56	1.76	0.61	1.82	0.44	1.17	0.76	1.80
**Problem with sociability**	0.51	1.76	0.48	2.03	0.75	1.56	0.60	2.28
**Problem with Submissiveness**	0.77	2.19	0.90	2.38	0.70	1.75	0.79	2.53
**Problem with intimacy**	0.62	1.77	0.66	1.57	0.92	1.49	0.75	1.73
**Problem with taking responsibility**	0.44	2.39	0.48	2.53	0.51	1.98	0.43	2.60
**Problem with controlling**	0.63	1.69	0.71	1.72	0.47	0.01	0.69	1.68
**Interpersonal problems**	2.14	11.44	2.04	12	2.55	8.98	2.43	12.84
**Psychological flexibility**	7.51	48.50	6.46	50.66	6.05	42.80	7	50.60

To see if the data is normal, Kolmogorov-Smirov test and to see the equality of variances, Leven test, and for studying the equality of correlation between variables, box's M test was administered. For assessing the degree of effectiveness of the intervention on all the dependent variables, Wilk's Lambda test was used. The results of Leven test for the equality of variances showed that in all the studied variables, there was equality of variances. The results of multivariate analysis of covariance showed that the linear combination of the studied variables had a significant difference regarding the two groups (P<0/001, F=26.49, and Wilk's Lambda=0.22). For determining the difference between the two groups regarding each variable, multivariate analysis of covariance was done whose results are presented in Table 2.

**Table 2:** The results of the multivariate analysis of covariance of interpersonal problems and its components and psychological flexibility

Source/Index	SS	df	F	P	Effect Size	Statistical Power
**Problem with Assertiveness**	1.208	1	9.17	0.006	0.29	0.82
**Problem with sociability**	0.82	1	5.90	0.02	0.21	0.64
**Problem with Submissiveness**	1.64	1	12.01	0.002	0.35	0.91
**Problem with intimacy**	2.66	1	9.23	0.006	0.29	0.82
**Problem with taking responsibility**	1.24	1	11.29	0.003	0.33	0.89
**Problem with controlling**	2.88	1	9.14	0.006	0.30	0.83
**Interpersonal problems**	76.19	1	68.76	0.0001	0.71	0.99
**Psychological flexibility**	91.81	1	39.71	0.0001	0.61	0.99

Regarding Table 2, the results of multivariate analysis of covariance showed that there is a significant difference between individuals with social anxiety disorder in the experimental and control groups. In other words, ACT reduced interpersonal problems and their components and increased psychological flexibility in the experimental group in comparison to the control group in the post-test stage. The effect of this intervention therapy on the decrease of interpersonal problems was 71% i.e. 71% of the total scores of the residual variance is related to the effectiveness of the intervention therapy. The statistical power of 100% indicates high statistical precision. Furthermore, the results of multivariate analysis of covariance revealed that there is a difference between female students with social anxiety disorder in the experimental and control groups regarding psychological flexibility (P<0/0001, F=39.71). Therefore, ACT was effective on the increase of psychological flexibility in the experimental group in the post-test. The effect size in the post-test was 0/61.

## 4. Discussion and Conclusion

The present study aimed to assess the effectiveness of ACT on the interpersonal problems and psychological flexibility in girls with social anxiety. The findings of this research showed that ACT reduces interpersonal problems of the subjects in the experimental group in comparison to the control group. Although no researches have been found on the effectiveness of ACT on interpersonal problems, the researches done on this variable in other therapies are in line with the results of this research (Borge, Hoffart, Sexton, Clark, Markowitz, & McManus, 2008; McEvoy, Burgess, & Nathan, 2013). It can be explained that ACT teaches identification of judgments, evaluations, predictions, and introducing them as the internal mind and world and defusion and lack of fusion with them and draws individuals into values, and valuable goals in life. On the other hand, teaching effective interpersonal communication and assertiveness methods in the therapy protocol helps individuals with solving problems resulting from not learning how to make relationships and communicate and indecisiveness.

The findings of the research also indicated that there is a significant difference between the scores of the experimental group and the control group regarding psychological flexibility. In other words, ACT increases psychological flexibility in the subjects of the experimental group than in the control group. This result is consistent with other researches (Hayes & Strosahl, 2010). The explanation for this finding could be that although in this therapy exposure inside the session was not used, the exercises of behavioral commitment necessarily involves exposure to social situations outside the session. Defusion techniques and acceptance reduce the rate of irritation and annoyance of these situations for the patients. Although this therapy does not directly target the frequency and content of the mind of a person with social anxiety, the reduction of anxiety in social situations as a result of defusion and acceptance techniques, detailed discussions about individuals’ values in social relations, individuals’ goals, and explanation, clarification, and stipulation of values will all lead to the reduction of thoughts and the reduction of avoidance of the person from social situations.

In this therapy, instead of focusing on exposure, increasing the individual's tendency to experience an inner event as it is highlighted. The aim here is to help the individual to experience a thought which is due to schemes just as a thought and instead of responding to it which is generally avoiding social situations and friends, the person learns to do what is important to him in life and what is in line with his values i.e. a thought about what people will think in their relations with me or other thoughts in relations with others is not a problem but the main problem is the person's effort to respond to these thoughts and eventually avoiding the situations in which the person feels uncomfortable in and feels unrest or has thoughts about his schemes about making relationships and communicating with others.

In fact, the aim of this therapy was to increase one's behavioral repertoire when facing terrifying events which are stressful and annoying situations that he experiences in making relationships and communicating with others and this is called psychological flexibility.

With respect to the background and previous researches, the results of the present research show that this therapy, in comparison to other therapies, can be considered as the desirable therapeutic choice and it can provide a significant improvement by its processes. More research is needed to determine whether the processes that underpin ACT are different from available supported therapies. If ACT is something different, more research is needed to identify its final effect. Any research is inevitably faced with restrictions that note to change findings with regard to those limitations. Gender (using female subjects), no follow up to evaluate the effectiveness of the therapy in the long-term and convenience sampling were the limitations of this research. Thus, the generalization of the results should be cautious. It is suggested that future studies increase the generalizability of the results using both genders of boys and girls, and follow-up stage. Furthermore, using ACT to increase individuals’ repertoire of behaviors and to movie in line with valuable life routes and increase the quality of people's lives is recommended.
